# Supplementary material for: Inhibition of Adipose Tissue Beiging by HIV Integrase Inhibitors, Dolutegravir and Bictegravir, Is Associated with Adipocyte Hypertrophy, Hypoxia, Elevated Fibrosis, and Insulin Resistance in Simian Adipose Tissue and Human Adipocytes
Source: Cells. 2022 Jun 4;11(11):1841. doi: 10.3390/cells11111841 (PMC9180037; doi:10.3390/cells11111841)
Supplement: Supplementary file 1 [file cells-11-01841-s001.zip › cells-1725840-supplementary.pdf]

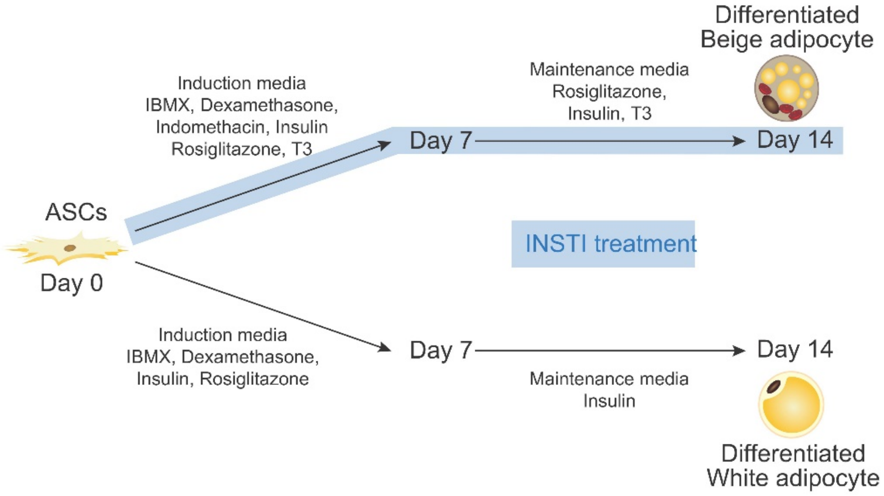

Supplementary Figure S1: Differentiation protocol and treatment of ASCs.

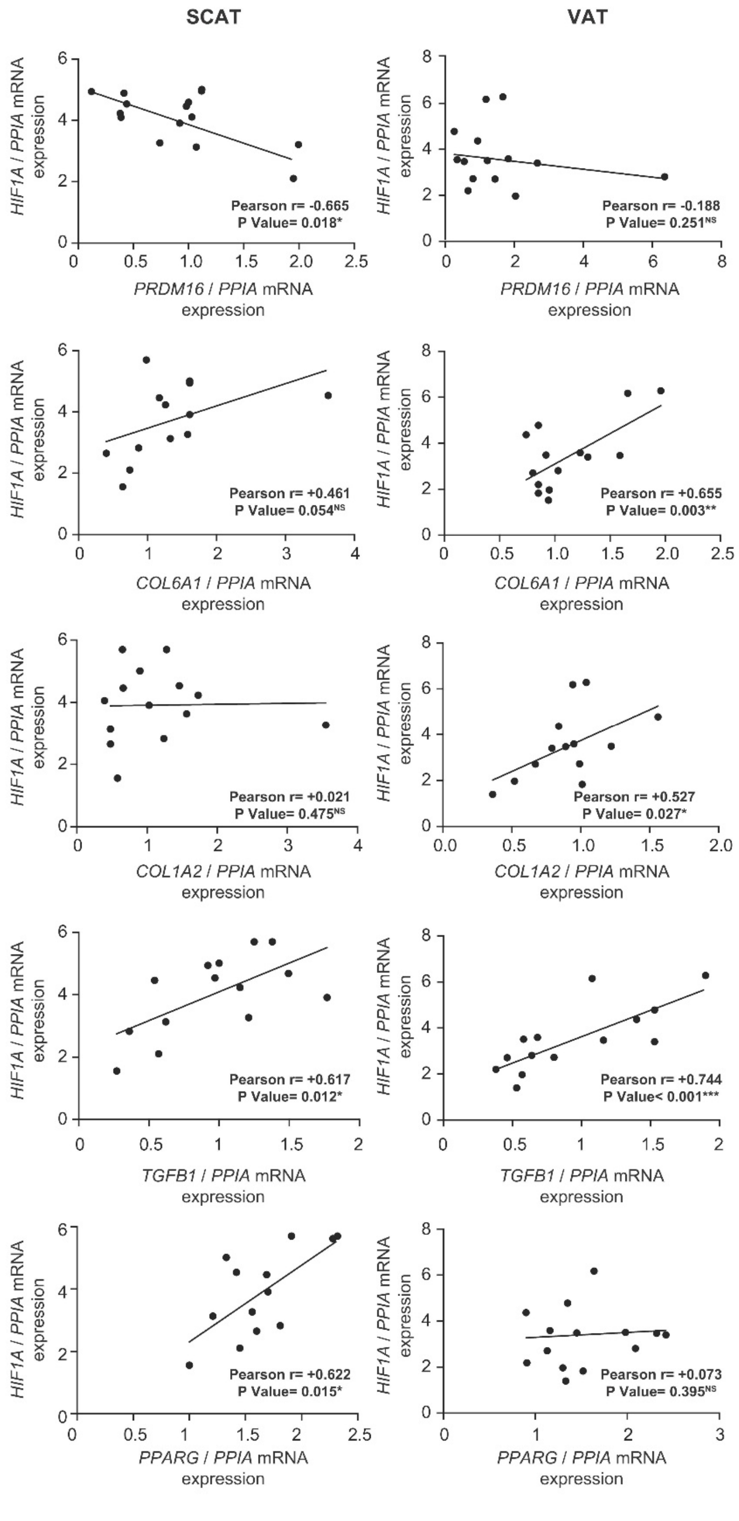

Supplementary Figure S2: Scatters plots of the correlation between the mRNA levels of *HIF1A* and *PRDM16*, *COL6A1*, *COL1A2*, *TGFB1* and *PPARG* in SCAT (left panel) and VAT (right panel). Correlations were determined using PCR data obtained using SCAT and VAT from 6control uninfected and untreated macaques (Ctrl) and 8 SIV-infected and ART-treated macaques (SIVART).

Supplementary Table S1. Sequences of oligonucleotides used for PCR on macaque AT samples.

Gene	forward primer	reverse primer
PPARG	cagtggggatgtctcataa	cttttggcatactctgtgat
COL1A2	ctggagaggctggtactgct	agcaccaagaagaccctgag
COL6A1	gaagagaaggccccgttg	cggtagcctttaggtccgata
TGFB1	actactgcttcagctccacg	agaagttggcatggtagccc
FN1	ctggccgaaaatacattgtaaa	ccacagtcgggtcaggag
HIF1A	ttttcaagcagtaggaatt	gtgatgtagtagctgcatg
PRDM16	tggctgcttctggactca	atattatttacaacgtcaccgtcact
TMEM26	ttgcaccatgagacccagt	tgctggtattctgtgatgttcc
PPIA	atgctggacccaacacaaat	tctttgactttgcaaacacc

Supplementary Table S2. Sequences of oligonucleotides used for PCR on human Adipocyte-derived ASCs.

Gene	forward primer	reverse primer
PPARG	cagtggggatgtctcataa	cttttggcatactctgtgat
SREBF1c	ggagggtagggccaacggcct	catgtcttcgaaagtgcaatcc
FABP4	gctttgccaccaggaaagtg	atgacgcgattccaccaccag
FASN	caggcacacacgatggac	cggagtgaatctgggttgat
SCD1	cctagaagctgagaaactggtga	acatcatcagcaagccaggt
TMEM26	ttgcaccatgagacccagt	tgctggtattctgtgatgttcc
CD137	agctgttacaacatagtaggccac	tcctgcaatgatcttgtcctct
PRDM16	tggctgcttctggactca	atattatttacaacgtcaccgtcact
CITED1	gagatggaaccatccgcaca	tcccgaggaactagtgggag
FGF21	gccttgaagccgggaagttatt	gtggagcgatccatacaggg
UCP1	ctggacacggccaaagtc	ggacacctttatacctaataacactgg
COL1A1	gggattccctggacctaaag	ggaacacctcgctctcca
TGFB1	gagcctgaggccgactacta	gggttcaggtaccgcttctc
COL1A2	ctggagaggctggtactgct	agcaccaagaagaccctgag
COL6A1	gaagagaaggccccgttg	cggtagcctttaggtccgata
ACTA2	cctatccccgggactaagac	aggcagtgctgtcctcttct
DDR1	aacaattcctctccggcactg	catgagggcaatgatgagcag
HIF1A	ttttcaagcagtaggaatt	gtgatgtagtagctgcatg
LOX	gggaatggcacagttgtca	acttgctttgtggccttcag
PPIA	atgctggacccaacacaaat	tctttgactttgcaaacacc

Supplementary Table S3. Summary comparing the impact of SIV-infection, ART-treatment alone and the combined effect of SIV infection and ART (SIVART) in SCAT and VAT from macaque studies. Adapted from the present study, and from results of our previous studies [3, 15].

	**TASC**			TAV		
	**SIV**	**ART**	**SIVART**	SIV	ART	SIVART
PPARG Expression	↓↓	↑↑↑	↑	↓	↑↑	↑
Fibrosis index	↑↑↑	↑↑↑	↑↑	↑↑	↑↑	↑↑
COL1A2 Expression	↑↑↑	↑↑	↑↑	↑↑	=	↑
COL6A1 Expression	↑↑	↑↑	↑↑	↑	=	↑
